# Bilateral single-port thoracoscopic extended thymectomy for management of thymoma and myasthenia gravis: case report

**DOI:** 10.1186/s13019-016-0547-3

**Published:** 2016-11-22

**Authors:** Francesco Paolo Caronia, Alfonso Fiorelli, Ettore Arrigo, Sebastiano Trovato, Mario Santini, Attilio Ignazio Lo Monte

**Affiliations:** 1 Thoracic Surgery Unit, Istituto Oncologico del Mediterraneo, Catania, Italy; 2 Thoracic Surgery Unit, Second University of Naples, Piazza Miraglia, 2, I-80138 Naples, Italy; 3 Department of Surgery, Università degli Studi di Palermo, Palermo, Italy

**Keywords:** Thymoma, Uniportal, Thoracoscopy, Myasthenia gravis, Bilateral, Case report

## Abstract

**Background:**

Video-assisted thoracoscopy is become a widely accepted approach for the resection of anterior mediastinal masses, including thymoma. The current trend is to reduce the number of ports and minimize the length of incisions to further decrease postoperative pain, chest wall paresthesia, and length of hospitalization. Herein, we reported an extended resection of thymoma in a patient with myasthenia gravis through an uniportal bilateral thoracoscopic approach.

**Case presentation:**

A 74 years old woman with myasthenia gravis was referred to our attention for management of a 3.5 cm, well capsulate, thymoma. All laboratory and cardio-pulmonary tests were within normal; thus, she was scheduled for thymoma resection through an uniportal bilateral thoracoscopic approach. Under general anaesthesia and selective intubation, the patient was placed in a 60° right lateral decubitus. A 3 cm skin incision was performed in the fourth right intercostal space and, through that a 30° video-camera and working instruments were inserted without rib spreading. After complete dissection of the thymus and mediastinal fat, the contralateral pleura was opened, and, through that the specimen was pushed into the left pleural cavity. Then, the patient was placed in the left lateral decubitus. Similarly to the right side procedure, a 3-cm incision was performed in the fourth left intercostal space to complete thymic dissection and retrieve the specimen. No intraoperative and post-operative complications were found. The patient was discharged four days later. Pathological examination revealed a type A thymoma (Masaoka stage I). No recurrence was found at 18 months of follow-up

**Conclusions:**

Bilateral single-port thoracoscopy is an available procedure for management of thymoma associated with myasthenia gravis. The less post-operative pain, the reduction of hospital stay and the better esthetic results are all potential advantages of this approach over traditional technique. Obviously, our impression should be validated by larger studies in terms of long-term oncological outcomes.

**Electronic supplementary material:**

The online version of this article (doi:10.1186/s13019-016-0547-3) contains supplementary material, which is available to authorized users.

## Background

Surgery using a standard or partial sternotomy is still the management of choice for management of thymoma. Over the years, standard multi-port Video Assisted Thoracoscopic Surgery (VATS) has become with the recent advances in instrumentation and techniques a feasible strategy for management of lung and mediastinal diseases [1–7]. The current trend is to reduce the number of ports and minimize the length of incisions to further decrease postoperative pain, chest wall paresthesia, and length of hospitalization [8–11]. Herein, we reported the resection of thymoma in a patient with Myasthenia Gravis (MG) through uniportal bilateral VATS approach.

## Case presentation

A 74 years old woman with MG was referred to our attention for management of thymoma. Chest computed tomography scan showed a 3.5 cm well capsulated mass within anterior mediastinum without invasion of adjacent structure. No other lesions were found. All laboratory and cardio-pulmonary tests were within normal, thus, she was scheduled for surgery. Plasmapheresis was performed preoperatively over a period of three days. She signed a written informed consent for the operation and was aware that her data could be used for scientific purpose only.

**Fig. 1:**
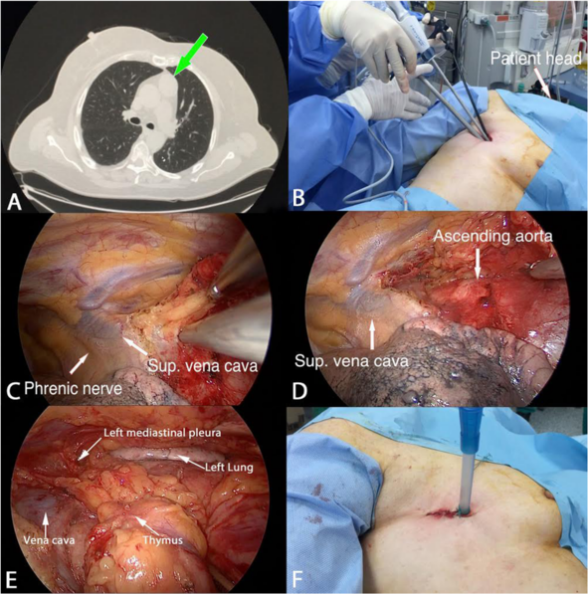
The chest computed tomography scan showed a 3,5 cm thymoma (Part **a**). Patient was placed in left lateral decubitus position, with the surgeon and the assistant standing on the posterior side (Part **b**). Dissection was landmarked by the superior border of phrenic nerve (Part **c**). The thymus was retracted superiorly and medially; the superior vena cava and the ascending aorta were skeletonised (Part **d**). Right side thymic and perithymic fatty tissue dissection was completed and left lung visible (Part **e**). Closure of right incision with a chest drainage (Part **f**)

Under general anaesthesia and selective intubation, the patient was placed in a 60° right lateral decubitus with a roll placed under the shoulder and the ipsilateral arm wrapped by a sterile stockinet and maintained parallel to the body. A 3 cm skin incision was performed in the fourth right intercostal space and through that a 30° camera and working instruments were inserted without rib spreading. The dissection started from the peri-cardiophrenic angle and continued cranially along the anterior border of the phrenic nerve. The thymus was then mobilized from the surrounding fat tissue and vena cava. Following, the contralateral pleura was opened, and, through that the thymus was pushed into the left pleural cavity. A single 24 fr chest tube was inserted through the same incision within right costo-phrenic angle. Figure 1 summarized the right side procedure.

**Fig. 2:**
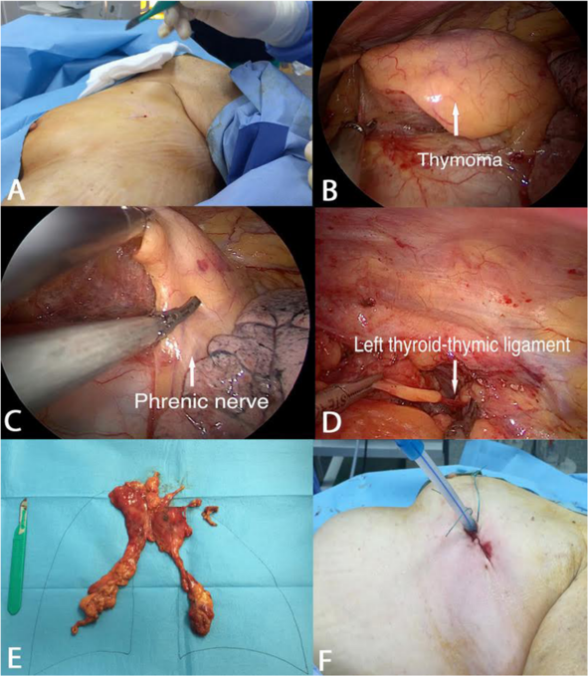
A 4 cm length incision was performed at the level of the fourth intercostal among the anterior and middle axillary line (Part **a**). A well capsulated thymoma is visible (Part **b**). Thymic dissection is conducted parallel to left phrenic nerve (Part **c**). The left thyroid-thymic ligament is shown before its section (Part **d**). The "en bloc specimen consisting of thymus, peri-thymic and peri-cardiophrenic fatty tissue (Part **e**). Closure of left incision with a chest drainage (Part **f**)



The patient was then placed in the left lateral decubitus. Similarly to the right side procedure, a 3-cm incision was performed in the fourth left intercostal space. The mediastinal pleura was dissected along the anterior border of the phrenic nerve. The thymus was mobilized to expose the thymic veins, the innominate vein and the thyroid-thymic ligaments. Then, the mediastinal fat was fully dissected from the phrenic nerve, the innominate vein, the aorto-pulmonary window, the aorto-caval groove, and the peri-cardiophrenic angle. After completing thymic dissection, the specimen was retrieved through the same incision and a chest tube was then inserted. Figure 2 summarized the left side procedure. No intraoperative and post-operative complications were found. The patient was discharged four days later. Pathological examination revealed a type A thymoma (Masaoka stage I). At 18 months of follow-up, the patient did not present recurrence. The main steps of the procedure are summarized in Additional file 1: Video 1.

**Additional file 1: Video 1.** Video edited the main steps of operation on the right and on the left side.


Over the past 10 years, VATS is become a widely accepted approach for the resection of anterior mediastinal masses, including thymoma. Numerous studies [5–7] confirmed that VATS thymectomy, when an *en bloc* resection of the tumor is achieved, has the same oncological results of standard sternotomy but with the advantages of less post-operative pain, better cosmesis and preservation of pulmonary function. The most popular approach is three-port VATS. Since 2004, single-incision thoracoscopic surgery has been reported but for a time its use was limited to pleural disease or wedge resection, until Gonzalez-Rivas et al. [8] described their first experiences of single-port thoracoscopic lobectomy. However, there are very few reports about single-port mediastinal tumour resection. We firstly reported the resection of thymic hyperplasia for management of MG using an uniportal bilateral VATS approach [9]. After that, other authors [1, 10, 11] confirmed the feasibility of our experience. Scarci et al. [4] reported a case series of 11 uniportal VATS thymectomies performed with an unilateral VATS approach. Wu L et al. [10] published thoracoscopic extended thymectomy through a single subxiphoid incision in 6 consecutive patients. Wu CF et al. [11] reported single-port VATS resection of thymoma, mediastinal cystic lesions and posterior mediastinal tumours.

From a technical point of view, the patient position and the bilateral approach are the main differences of our compared to other techniques. Instead of 30° semisupine or semiprone position proposed by Wu CF et al. [11], we preferred to place the patient in a 60° right lateral decubitus position with the ipsilateral arm maintained parallel to the body to facilitate the dissection of mediastinal fat dissection in a difficult anatomical region as the cardio-phrenic area. Conversely to unilateral approach proposed by Scarci et al. [4] and Wu L et al [10], we performed a bilateral approach that allowed to carry out an *en bloc* dissection of thymus, peri-thymic and bilateral peri-cardiophrenic fatty tissue. It is crucial to prevent tumor and MG recurrences. Compared to our first case [10], in the current the presence of thymoma made the procedure more challenging. Thus, we started the dissection from the right side where the thymoma was less protruding to the mediastinum in order to minimize the risk of capsular breakage and tumour seeding. Then, the left approach allowed the complete resection of the tumor and its removal under endoscopic vision.

The patient selection is critical for the successful of the procedure. Our minimally invasive approach is indicated for resection of small and early stage thymomas, while, in line with other experiences [12, 13], conventional open sternotomy remained the strategy of choice for management of thymomas > 4 cm and/or advanced stage thymomas to avoid capsule injury and/or lesion of vital structures during dissection. Ye et al. [13] reported a conversion to sternotomy in 4/125 patients undergoing VATS resection for thymomas having a mean size of 40 ± 8.2 mm because of injury to the innominate vein. The presence of MG associated to thymoma is not a main contraindication for VATS. However, in these cases a bilateral approach is the preferred choice in order to carry out the “*en bloc”* resection of thymoma and of bilateral mediastinal fat for preventing thymoma and MG recurrences. The procedure should be performed under general anesthesia and selective-lung ventilation. However, the use of CO2 gas insufflation could be an additional strategy to improve the operative field vision especially when complete lung exclusion is not available.

## Conclusions

Bilateral single-port thoracoscopy is an available procedure for management of thymoma associated with MG. The less post-operative pain, the reduction of hospital stay and the better esthetic results are all potential advantages of this approach over traditional technique. Obviously, our impression should be validated by larger studies in terms of long-term oncological outcomes.

## Abbreviations

MG: Myasthenia gravis
VATS: Video assisted thoracoscopic surgery
